# Intradural Intramedullary Spinal Cord Glioblastoma: A Case Report

**DOI:** 10.7759/cureus.43580

**Published:** 2023-08-16

**Authors:** Pritee Shrestha, Tara Eineichner, Brittany Wilson, Naomi S Lam

**Affiliations:** 1 Internal Medicine, MercyOne North Iowa, Mason City, USA; 2 Surgery, Des Moines University, Des Moines, USA; 3 Internal Medicine, Des Moines University, Des Moines, USA

**Keywords:** primary brain tumor, primary spinal glioblastoma multiforme, central nervous system neoplasms, central nervous system, chemo-radiation

## Abstract

Primary spinal cord glioblastoma multiforme (GBM) remains uncommon and typically affects males and patients during their fifth decade of life. Our case demonstrates a 77-year-old woman who initially presented with right arm paresthesia and limited range of motion and was subsequently diagnosed with primary spinal cord GBM. Our case illustrates an atypical and nonspecific neurological presentation highlighting that spinal cord GBM can have a more indolent course, unlike what current literature suggests. It also emphasizes the importance of considering a multimodal approach when managing atypical neurological symptoms and considering an early intervention, including magnetic resonance imaging, to rule out occult neoplasm in an appropriate clinical setting, thus preventing delay in the diagnosis. This case further emphasizes the role of molecular biomarkers of tumors, including isocitrate dehydrogenase mutation as well as methylguanine-DNA methyltransferase promoter methylation status, that can independently guide and affect the treatment outcomes in this patient population.

## Introduction

Glioblastoma multiforme (GBM) is a highly malignant and aggressive primary central nervous system cancer. While GBM is most common in older people, it can occur at any age, it mostly occurs in the fifth decade of life [1]. The age of onset and its rarity in general makes our case of primary spinal cord GBM particularly unique. Compared to the typically rapidly progressive nature of these tumors, the primary spinal cord GBM, in this case, may have a longer, more docile course before diagnosis, making it an even more unusual presentation. Surgical resection, radiation therapy (RT), chemotherapy, and targeted therapy are the usual treatment for GBM; however, its aggressive nature frequently leads to limited success, and disease progression continues [2]. This case illustrates the significance of considering spinal cord GBM when determining the differential diagnosis of spinal cord malignancies, even in individuals over the age of 60. Its aggressive course demands further research and the discovery of novel treatments to improve patient outcomes.

## Case presentation

**Figure 1:**
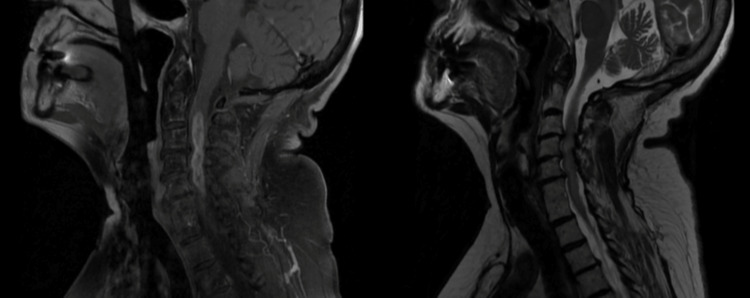
The left image shows sagittal T1 cervical spinal cord imaging with contrast and the right image shows sagittal T2 imaging of the cervical spinal cord.

A 77-year-old woman presented with acute right arm weakness, paresthesia, and limited range of motion in addition to worsening neck pain and shoulder ache. Her pertinent past medical history included insulin-dependent diabetes mellitus, diabetic neuropathy, coronary artery disease, hypertension, and migraines. The rest of the review of systems was negative, including trauma, lower extremity weakness or numbness, speech, swallowing, chest pain, dizziness, and migraines. Her cervical spine and AC joints showed severe osteoarthritis on a radiograph. Her symptoms were initially associated with rotator cuff tendinopathy, but her symptoms did not improve with physical therapy and a course of steroid treatment. Due to continued symptoms, magnetic resonance imaging (MRI) of the shoulder and cervical spine was obtained. A cervical spine MRI showed a long ovoid, lobulated intramedullary mass with solid and rim enhancement in the cervical cord from C2 to C5-C6 (Figure 1). The solid mass was 5.3 cm long and 8 × 14 mm wide. Vasogenic edema with central necrosis spanned the C2-C6 levels. The mass expanded into the subaxial spine with eccentricity to the right caudally, staying midline at C2-C3. Brain MRI and computed tomography (CT) of the chest, abdomen, and pelvis with contrast were unremarkable.

Neurosurgery was consulted. Further review of history revealed that the patient had experienced progressive gait changes and was veering toward the right side over the past two years. Additionally, the patient reported back pain with increasing episodes of bowel and urinary incontinence over the duration. Upon re-evaluation of her physical examination, the patient exhibited spastic paresis of the right upper limb with diffuse weakness: 3/5 of the deltoid, 1/5 of the bicep, 4/5 of the tricep, and 4/5 in hand grip. The right lower limb scored 4/5 for hip flexion and extension, but the remainder of the muscle strength was full. There was also decreased sensation in a non-dermatomal fashion in the left upper and lower limb. The deep tendon reflexes of the knee were +3 bilaterally, but the remainder of all reflexes were +1. Positive Hoffman’s and Romberg’s signs with an unstable gait were also noted. Given the patient’s progressive symptoms and the location of the tumor, immediate surgical management was discussed for definitive treatment and further pathological analysis. The patient agreed to undergo surgery despite the high risk of quadriplegia or quadriparesis. The patient underwent surgery to remove the C2-C6 intramedullary spinal cord tumor two months after the initial MRI. C2-C6 laminectomy and Medtronic Infinity posterior cervical instrumented fusion were performed. C2-C6 excision removed the tumor while monitoring spinal cord function. The final intraoperative neuromonitoring indicated stable motor-evoked potentials but loss of right arm and leg somatosensory-evoked potentials. An immediate postoperative cervical spine MRI without contrast showed a persistent tumor at C2-C3 with T2 hyperintensity and enhancement.

The final biopsy report of the specimen showed a highly cellular, infiltrating astrocytoma composed of cells with large irregular nuclei and moderate-to-abundant eosinophilic cytoplasm. Mitotic activity (up to eight mitoses in 10 high-power fields), focal microvascular proliferation, tumor thrombosis, and necrosis were present. Immunobiological stains were performed on paraffin-embedded tissue at Mayo Clinic using antibodies to IDHI-R132H, ATRX, 1-13 K27M, and H3 K2rrna3. Mutant IDHI (IDH1-R132H) and ATRX analysis revealed that the glioblastoma was negative for the mutant IDH1-R132H protein, essentially excluding the most common IDH mutation. The expression of ATRX was retained. H3 K27M was negative. K27me3 expression was retained in the tumor cells. The methylguanine-DNA methyltransferase (MGMT) promoter methylation study was negative. WHO grade 4 spinal cord tumors consistent with IDH wild-type (IDH-wt) glioblastoma was diagnosed.

Following the surgery, the patient was transferred to the intensive care unit and had a good recovery during her hospital stay. Although there was a slight improvement in her right-sided hemiparesis, her hand grip remained at 2/5, foot plantarflexion and dorsiflexion were at 3/5, and bicep strength was at 1/5, while triceps remained at 4/5. The cervical spine was treated with 50 Gy in 25 portions with volumetric-modulated arc therapy and intensity-modulated radiation therapy. Oral temozolomide was given at 75 mg/day for five days per week during concomitant treatment. Neurologic symptoms improved during adjuvant chemoradiation, including right leg mobility and neck pain. Despite these findings, the patient continued to have significant right-sided hemiparesis with severe right upper extremity weakness and difficulty in standing up due to right lower extremity weakness and decreased balance. The clinical course was complicated by a urinary tract infection requiring hospitalization. After recovery, the patient continued to have outpatient oncology follow-up. During ongoing chemotherapy and RT, the patient passed away post-discharge.

## Discussion

Gliomas are glial or precursor cell-derived primary central nervous system (CNS) cancers. Astrocytic cancers include astrocytoma, GBM, oligodendroglioma, ependymoma, mixed glioma, malignant glioma, not otherwise specified (NOS), and a few rarer histologies [1]. According to the WHO, GBM is the most frequent malignant CNS tumor and the most aggressive diffuse astrocytic glioma, grade IV. Although a multidisciplinary approach that includes surgery, radiotherapy, systemic chemotherapy and/or targeted therapy, and supportive care has improved treatment options, the aggressive nature of this tumor continues to portend a poor prognosis and rare long-term survival [2]. At five years post-diagnosis, only 5.8% survive. Median survival is 14-15 months [2,3]. GBM occurs in less than 10 per 100,000 people worldwide [3]. It can occur at any age but is most common in people aged 75-84, with a median age of 65 at diagnosis [1]. GBM is most common in non-Hispanic whites with the lowest prevalence (40%) in American Indians or Alaska Natives [2].

The underlying cause(s) of GBM remains unclear. Currently, no identifiable carcinogenic factors have been established [4]. Exposure to high levels of ionizing radiation is hypothesized to be a potentially modifiable risk factor for GBM [3-5]. The brain stem, cerebellum, and spinal are rare sites of GBM, but the cerebral hemispheres, especially the supratentorial region, are common sites [5]. GBM is aggressive because neoplastic cells can migrate great distances along neuropil structures. These tumors have pleomorphic cells with tiny, big, or gliosarcoma patterns histologically [6]. GBM shows mitotic activity, intravascular microthrombi, necrosis, pseudo palisading, and microvascular growth.

The four transcriptional subtypes of classical, mesenchymal, proneural, and neural GBM can also be categorized molecularly [5,6]. The loss of PTEN and cyclin-dependent kinase inhibitor 2A (CDKN2A), as well as the deletion of p16INK4A, are characteristics of classical GBM [5]. The mesenchymal subtype is linked to modifications and deletion of the TP53, neurofibromin-1 (NF-1), and CDKN2A [5]. PDGF, IDH1/IDH2, p53, phosphoinositide-3-kinase catalytic subunit alpha (PI3KCA), and phosphoinositide-3-kinase regulatory subunit 1 (PI3KR1) mutations all belong to the proneural subtype [6]. There is no known genetic defect linked to neural GBM [5].

In particular, IDH-wt glioblastoma that was diagnosed in our patient constitutes the most common and aggressive GBM subtype [7]. By definition, these glioblastomas lack mutations in IDH1/2 and are classified as IDH-wt glioblastoma per the 2021 WHO revision. Some of the primary characteristics of these types of glioblastoma are diffusely infiltrative and cellular, pleomorphic, and high mitotic activity associated with necrosis [8]. This subtype of glioblastoma demonstrates a complex molecular profile involving alterations including amplified epidermal growth factor receptor (EGFR), p53 mutations, loss of PTEN and CDKN2A function, RB1 alterations, disruptions in the PI3K-AKT-mTOR pathway, TERTp mutations, and mutations in NF1 leading to unchecked cell growth and enhanced cell survival and enhancing the aggressive nature of the tumor. Other associated alterations in the function of CDK4, CDK4/MDM2, CDKN2A, 19+/20+, and MGMT are also involved [9].

CT and MRI are the two main imaging tests used on patients who are thought to have CNS malignancies [5,10]. To identify a mass and rule out pathology from other sources, such as bleeding, CT is typically performed during the presentation [10]. A midline shift can also be noticeable if there is significant accompanying edema [5]. Due to improved soft tissue differentiation over CT, MRI with T1-weighted, T1-weighted contrast-enhanced, T2-weighted, T2-fluid-attenuated inversion recovery, and gradient echo sequences is the gold standard imaging study and should be used if there are no MRI-specific contraindications [5]. GBM frequently exhibits central rim-enhancing lesions and surrounding white matter edema [5].

The usual appearance of these tumors on T1-weighted images is that of penetrating, enhancing lesions with hyperintense T2 signal and heterogeneous enhancement [11,12]. Spinal cord GBM also exhibits elevated blood volume, decreased N-acetyl aspartate, and increased choline/creatine ratio [13]. GBMs can form anywhere along the neuroaxis, but they are most frequently seen in the cerebral hemispheres’ supratentorial region [6]. Overall, only 10% of CNS cancers are spinal cord tumors, which are extremely uncommon [13]. Only 1.5% of all spinal cord tumors are primary GBMs [14]. Primary spinal cord GBM, in contrast to cranial GBM, typically manifests in male patients and has a very aggressive clinical course. Before serious neurological deterioration and death, patients may first have symptoms that are consistent with disc herniation. The subarachnoid area is easily accessible, allowing for quick leptomeningeal penetration and subsequent early brain metastases [15]. The average time to survival after diagnosis is 10-14 months.

Like in the case of our patient, neurological presentation due to the location of the tumor has been defined in the literature (Table 1). Although such motor and neurological symptoms associated with pain seem to be the most common presenting symptoms of GBM, the diagnosis of spinal cord GBM can be easily overlooked and missed due to its rarity.

**Table 1:** Neurological presentation of glioblastoma multiforme in different cases.

Authors	Patient description	Symptoms and presentation	Diagnosis
Yang et al. [14]	Case 1: a 48-year-old woman	Left arm numbness, mild cervical pain	Ring-enhancing lesion on MRI, hypointense on T1, hyperintense on T2 Diagnosis: GBM
Yang et al. [14]	Case 2: a 65-year-old male	Upper dorsal spine pain	Diagnosis: primary spinal cord GBM
Yang et al. [14]	Case 3: a 21-year-old female	Non-radiating lower back pain, weakness, unsteady gait, urine retention Pathology: neoplastic cells, vascular proliferation, necrosis, positive for glial fibrillary acidic protein, negative for IDH1	Diagnosis: giant cell GBM
Ginat and Schaefer [13]	Case 4: a 22-year-old female	Low back pain radiating to the left lower extremity, left lower extremity weakness MRI finding: intramedullary mass Histopathology: GBM, grade IV	Diagnosis: GBM

To date, the largest single-center study of primary spinal cord GBM was performed at Beijing Tsinghua Changgung Hospital and involved only 15 patients diagnosed between May 2015 and June 2019, emphasizing how infrequently these tumors occur [13]. The current case describes primary spinal cord GBM in a 77-year-old female. Additionally, this case describes a potentially longer, more indolent course before diagnosis. Primary spinal cord GBM is characterized by a highly aggressive clinical course following a short presenting history with overall survival of approximately 10-14 months [16]. In this case, the patient experienced symptoms of back pain, gait disturbance, and bowel and bladder incontinence for approximately two years before her initial presentation. This suggests primary spinal cord GBM may have a longer, subclinical stage followed by a transition to a more aggressive, rapidly progressive stage.

For the majority of patients with GBM, the current standard of care entails surgical resection, RT, and concurrent adjuvant chemotherapy using the alkylating drug temozolomide (TMZ) [17]. As we discuss the treatment of GBM, consideration of two important enzymes, namely, IDH and MGMT, is important. The presence of IDH mutations triggers epigenetic changes resulting in the loss of enzymatic activity of IDH and conferring a better prognosis. Along with this, MGMT promoter methylation suppresses MGMT and subsequently reduces the transcription of the enzyme that degrades TMZ [18,19]. Thus, both enzymes influence the response to TMZ treatment. Applying this to our case, the cumulative effect of retained wild-type IDH and lack of methylation in the MGMT gene in our patient could potentially have rendered an unfavorable prognosis.

Primary RT is used to treat cancers that are inoperable [4]. Better clinical results have lately been achieved with a non-invasive technology known as the tumor-treating field, Optune, Novocure GmbH, and TMZ [5]. According to current guidelines, patients who have a fair prognosis and are less than 70 years old should receive regular RT along with concurrent adjuvant TMZ and alternating electric field therapy, regardless of the MGMT methylation status of the lesion as tumors without MGMT promoter methylation have a poor prognosis and are resistant to alkylating therapy, just like in the case of our patient [13]. Patients with excellent prognoses who are older than 70 years old should receive hypofractionated or conventional RT along with concurrent and adjuvant TMZ and alternating electric field therapy [20]. Patients may receive up to six TMZ maintenance rounds following their initial treatment. In more common isoforms that overexpress EGFR and PTEN, the EGFR-inhibitor/tyrosine kinase inhibitor Erlotinib shows minimal effectiveness; similarly, numerous more targeted treatments for GBM have failed during development. These findings highlight complex pathophysiology and provide a possible insight into treating GBM. Finally, while complete cord amputation for lower spinal cord lesions has been proposed which is a benefit compared to treatment of aggressive intracranial glioblastoma, this was not a feasible option in our patient due to the high cervical location.

## Conclusions

This case presents a rare case of primary GBM in a 77-year-old woman, which is remarkable as this type of tumor usually affects patients in the fifth decade of life. Further examinations revealed a grade 4 GBM in the cervical cord, although the patient’s symptoms initially appeared to be rotator cuff tendinopathy. Thus, due to its rarity, GBM in the spinal cord can easily be missed as a differential which can ultimately delay timely intervention. Despite comprehensive treatment strategies comprising surgery, RT, chemotherapy, and targeted therapy, GBM remains a highly aggressive and malignant CNS tumor with poor overall survival. Unlike other primary spinal cord GBM cases, which often advance quickly, our patient had a longer, more gradual course before diagnosis. This case highlights the significance of taking primary spinal cord GBM into account when making a differential diagnosis for elderly patients exhibiting neurological symptoms. To improve outcomes for individuals with GBM and other aggressive primary CNS tumors, this case emphasizes the need for providers to consider a multimodal approach when managing atypical neurological presentation in high-risk age groups including consideration of timely MRI to evaluate for occult neoplasm.
